# Safety and immunogenicity of a plant-produced Pfs25 virus-like particle as a transmission blocking vaccine against malaria: A Phase 1 dose-escalation study in healthy adults

**DOI:** 10.1016/j.vaccine.2018.08.033

**Published:** 2018-09-18

**Authors:** Jessica A. Chichester, Brian J. Green, R. Mark Jones, Yoko Shoji, Kazutoyo Miura, Carole A. Long, Cynthia K. Lee, Christian F. Ockenhouse, Merribeth J. Morin, Stephen J. Streatfield, Vidadi Yusibov

**Affiliations:** a Fraunhofer USA Inc. Center for Molecular Biotechnology, Newark, DE 19711, USA; b Laboratory of Malaria and Vector Research, National Institute of Allergy and Infectious Diseases, National Institutes of Health, Rockville, MD 20852, USA; c PATH’s Malaria Vaccine Initiative, Washington, DC 20001, USA

**Keywords:** Malaria, Transmission blocking vaccine, Pfs25, Virus-like particle, Plant-produced

## Abstract

Malaria continues to be one of the world’s most devastating infectious tropical diseases, and alternative strategies to prevent infection and disease spread are urgently needed. These strategies include the development of effective vaccines, such as malaria transmission blocking vaccines (TBV) directed against proteins found on the sexual stages of *Plasmodium falciparum* parasites present in the mosquito midgut. The Pfs25 protein, which is expressed on the surface of gametes, zygotes and ookinetes, has been a primary target for TBV development. One such vaccine strategy based on Pfs25 is a plant-produced malaria vaccine candidate engineered as a chimeric non-enveloped virus-like particle (VLP) comprising Pfs25 fused to the Alfalfa mosaic virus coat protein. This Pfs25 VLP-FhCMB vaccine candidate has been engineered and manufactured in *Nicotiana benthamiana* plants at pilot plant scale under current Good Manufacturing Practice guidelines. The safety, reactogenicity and immunogenicity of Pfs25 VLP-FhCMB was assessed in healthy adult volunteers. This Phase 1, dose escalation, first-in-human study was designed primarily to evaluate the safety of the purified plant-derived Pfs25 VLP combined with Alhydrogel® adjuvant. At the doses tested in this Phase 1 study, the vaccine was generally shown to be safe in healthy volunteers, with no incidence of vaccine-related serious adverse events and no evidence of any dose-limiting or dose-related toxicity, demonstrating that the plant-derived Pfs25 VLP-FhCMB vaccine had an acceptable safety and tolerability profile. In addition, although the vaccine did induce Pfs25-specific IgG in vaccinated patients in a dose dependent manner, the transmission reducing activity of the antibodies generated were weak, suggesting the need for an alternative vaccine adjuvant formulation.

This study was registered at www.ClinicalTrials.gov under reference identifier NCT02013687.

## Highlights

• Report of a Phase I clinical trial to assess a malaria transmission blocking vaccine.
• *P. falciparum* Pfs25 virus-like particle produced under cGMP in a plant-based system.
• The vaccine candidate displays an acceptable safety and tolerability profile.
• The vaccine candidate induced Pfs25-specific IgG in a dose dependent manner.
• However, low transmission reducing activity implies need for an improved formulation.

## 1 Introduction

Malaria is a mosquito-borne, life-threatening, infectious disease caused by *Plasmodium* parasites. According to the World Malaria Report 2016, about 212 million cases of malaria were reported worldwide in 2015, predominantly in sub-Saharan Africa and South-East Asia, causing approximately 303,000 deaths, mostly among African children under the age of 5 years. Of the five species of malaria parasites that infect humans, *Plasmodium falciparum* is responsible for the majority of deaths [1].

The spread of the disease in endemic regions can be reduced by the use of insecticide-treated bed nets and indoor residual spraying. Furthermore, antimalarial medicines can be used both prophylactically and for curative treatment. However, recurring drug resistance compromises the efficiency of both old and new antimalarial medicines [2]. Thus, effective vaccines for the control and prevention of malaria are urgently needed, as vaccination remains one of the most efficient and cost-effective methods for controlling infectious diseases. Presently, there is only one licensed malaria vaccine available for areas where *Plasmodium falciparum* is prevalent. Most research activities on vaccine candidates including the licensed vaccine, Mosquirix, have been focused on pre-erythrocytic and asexual stages of the parasite life cycle [3], [4], [5], [6], [7], [8], preventing the occurrence or multiplication of pathogenic asexual parasite forms [9]. In 2011, the Malaria Eradication Research Agenda Consultative Group on Vaccines set as a core goal that any malaria vaccine program needs to reduce transmission as well as morbidity [10]. These initiatives to eliminate/eradicate malaria have intensified the interest in developing transmission blocking (TB) vaccines (TBVs). TBVs aim to prevent sexual stage parasites ingested by female *Anopheles* mosquitoes from undergoing successful sporogonic development, thus preventing transmission from mosquito to a potential human host and subsequent spread of parasites in endemic populations. Identified targets of effective TB immunity are proteins expressed on the surface of gametocytes/gametes, zygotes and ookinetes, as well as mosquito encoded proteins in the mid-gut. For example, antibodies against the *Plasmodium* proteins Pfs25, Pfs28, Pfs48/45 or Pfs230 have been shown to block parasite transmission to mosquitoes [11], [12].

Pfs25, one of the primary targets for TBV development, is a member of a protein family characterized by the presence of epidermal growth factor (EGF)-like repeat motifs, numerous cysteine residues and a complex tertiary structure [13]. Therefore, it has been difficult to produce Pfs25 with accurate conformation in heterologous systems. Additionally, *Plasmodium* parasites lack the N-linked glycosylation machinery, and many *Plasmodium* proteins contain multiple potential glycosylation sites that are aberrantly glycosylated when expressed in any of the available eukaryotic hosts [14]. Despite these challenges, recent success has been achieved with recombinant versions of Pfs25 proteins produced in yeast that are emerging as prominent TBV candidates [15], [16], [17], [18], [19], [20], [21], [22], [23]; the leading candidate being a *Pichia pastoris* produced Pfs25 (PpPfs25H-A) chemically conjugated to the mutant, non-toxic ExoProtein A (EPA) of *Pseudomonas aeruginosa* [24], [25].

During the last two decades, several groups have demonstrated the potential of plants as a safe, cost-effective and highly scalable platform for production of recombinant vaccine antigens and therapeutic proteins [26], [27], including variants of the soluble, full-length Pfs25 antigen [28], [29]. Virus-like particles (VLPs) are a class of subunit vaccines that induce the strongest protective immunity [30]. Recently, a plant-based malaria vaccine candidate, Pfs25-CP VLP, which represents a chimeric non-enveloped VLP comprising Pfs25 fused to the Alfalfa mosaic virus coat protein (CP), has been engineered, transiently produced in *Nicotiana benthamiana* plants using a Tobacco mosaic virus (TMV)-based hybrid vector[31], purified and characterized [32]. Immunization of mice with one or two doses of this vaccine candidate adjuvanted with Alhydrogel® induced serum antibodies with complete TB activity persisting through the six-month study period [32], supporting the evaluation of Pfs25-CP VLP as a potential malaria TBV candidate.

Subsequently, this malaria vaccine candidate, named Pfs25 VLP-FhCMB, was produced in *N. benthamiana* at pilot plant scale under current Good Manufacturing Practice (cGMP) guidelines, and results on safety, reactogenicity and immunogenicity assessed in healthy adult volunteers are presented here.

## 2 Materials and methods

### 2.1 Study design

This was a Phase 1, single-center, open-label, non-randomized, dose-escalation clinical study conducted at the Optimal Research, Accelovance Inc. (Rockville, Maryland). The study protocol (FhCMB Pfs25-001) and the informed consent form were approved by the Western Institutional Review Board (WIRB, Olympia, WA; Protocol Number: 20131400). The study was conducted in accordance with the principles of the Declaration of Helsinki, the standards of Good Clinical Practice (as defined by the International Conference on Harmonization) and federal regulations. All participants provided written informed consent prior to screening and enrollment into the study.

The primary objective of this study was to assess the safety, reactogenicity and tolerability of the Pfs25 VLP-FhCMB vaccine delivered IM at doses of 2, 10, 30 and 100 μg of total protein in healthy adults 18–50 years of age. The secondary objective was to assess the immunogenicity of the candidate vaccine administered with Alhydrogel after three vaccinations. Immunogenicity was assessed by measuring IgG antibody titers against Pfs25 by enzyme-linked immunosorbent assay (ELISA) and transmission reducing activity (TRA) by standard membrane feeding assay (SMFA).

This study was registered at www.ClinicalTrials.gov under reference identifier NCT02013687.

### 2.2 Vaccine

The Pfs25 VLP-FhCMB vaccine, developed by Fraunhofer USA Center for Molecular Biotechnology (FhCMB), is a VLP containing the recombinant Pfs25 antigen from *Plasmodium falciparum* engineered to the Alfalfa mosaic virus CP and produced in hydroponically grown *N. benthamiana* plants using a recombinant *A. tumefaciens*-delivered, TMV-based hybrid vector. The Pfs25-CP fusion antigen was cloned, expressed in *N. benthamiana*, purified and characterized as reported previously [32]. The drug product contained 400 µg of total protein per mL in an aqueous formulation containing 50 mM sodium phosphate for IM administration and was aseptically filled and packaged at Walter Reed Army Institute of Research (WRAIR) Pilot Bioproduction Facility (Silver Spring, MD). Four total protein dose levels of the vaccine (2, 10, 30 and 100 μg per 0.5 mL) were formulated in the clinic on the day of administration with 0.3% (w/w) aluminium hydroxide gel (Alhydrogel®) (to give 0.75 mg aluminium per 0.5 mL dose) adjuvant (Brenntag Biosector, Frederikssund, Denmark).

### 2.3 Inclusion and exclusion criteria

Subjects were healthy, as determined by medical history, physical examination, vital signs, and clinical safety laboratory examination at baseline, male or female adults aged 18–50 years (inclusive) at first vaccination, who had met the enrollment criteria. Major exclusion criteria included women who were pregnant, lactating, or planning on becoming pregnant during the study period and subjects with a history of malaria or previous receipt of an investigational malaria vaccine, being seropositive for hepatitis B surface antigen (HBsAg), hepatitis C antibodies (anti-HCV) or human immunodeficiency. Subjects with a history of any severe allergic or anaphylactic type reaction to injected vaccines, a history of chronic or active immunodeficiency, autoimmune disease or use of immunosuppressive medications within 3 months prior to any planned vaccine dose, abnormal baseline clinical safety laboratory tests, or had received or planned to receive any other experimental drug/vaccine or licensed vaccine within 30 days prior to vaccination were also excluded.

### 2.4 Vaccination schedule

A total of 44 subjects were enrolled and sequentially assigned to one of four study vaccine groups to receive the Pfs25 VLP-FhCMB vaccine adjuvanted with Alhydrogel®, at the following vaccine doses: Group 1 (2 μg, 6 subjects), Group 2 (10 μg, 6 subjects), Group 3 (30 μg, 16 subjects) and Group 4 (100 μg, 16 subjects). Since this was a dose-escalation study to assess the safety, reactogenicity and immunogenicity of Pfs25 VLP-FhCMB vaccine with a targeted human dose of 30–100 µg, smaller numbers of subjects were assigned to the two lower dose groups. The vaccination schedule was started with the lowest dose group and progressed group by group to higher dose groups. Subjects received three doses of the vaccine on Study Days 0, 56 (±4 days) and 168 (±7 days). A total volume of 0.5 mL was injected per vaccination; vaccinations were administered in the deltoid muscle of the non-dominant arm. The safety and tolerability of each vaccine dose level were assessed before moving to the next dose level, using pre-specified halting/holding rules (defined in Section 2.5).

On Study Day 0, two naïve subjects of Group 1 received 2 μg of the Pfs25 VLP-FhCMB vaccine. If there were no immediate safety issues, the remaining four subjects of this group received the 2 μg dose of the vaccine on Study Day 1 (approximately 24 h after the first two subjects). Safety data was collected for 6 days following each group vaccination and was reviewed by the Principal Investigator and the Safety Monitoring Committee (SMC) to ensure that neither Stop nor Hold criteria had been met prior to the administration of the next higher dose. There was an approximate 14-day stagger between each group dose escalation with the same safety follow-up procedures and SMC review occurring after each dose escalation.

Since the TRA immunologic criterion for the Go decision (nine-month follow-up after the third vaccination, defined in Section 2.6) was not met (see Section 3.3), the follow-up period and duration of subject participation were six additional months for safety evaluation. The final scheduled blood draw (evaluating safety and immunogenicity) occurred on Study Day 252 with a telephone follow-up for safety on Study Day 336.

### 2.5 Safety assessments

Safety was assessed by recording baseline demographic information and medical history, monitoring vital signs, adverse events (AEs) and concomitant medications, and performing physical examinations and laboratory tests for hematology, chemistry and pregnancy. Visual assessments of the injection site were made prior to and 30 min post-injection on Study Days 0, 56 and 168, along with once each follow up visit.

Solicited general Treatment-Emergent AEs were fever, myalgia, chills, sweats, fatigue/malaise, arthralgia, headache, nausea, vomiting or diarrhea; their severity was graded as Grade 1, 2 or 3. Solicited local Treatment-Emergent AEs (injection site reactions) were pain, tenderness, erythema and induration/swelling; their severity was graded as mild, moderate or severe. Additional grading scales were applied to visible swelling or redness at the injection site (1 = 2.5–5 cm, 2 = 5.1–10 cm and 3 = >10 cm) and to fever (1 = 38.0–38.4 °C, 2 = 38.5–38.9 °C, 3 = 39.0–40 °C and 4 = >40 °C). Unsolicited Treatment-Emergent AEs were assessed for severity and graded as 1 = mild, not interfering with routine activities, minimal level of discomfort; 2 = moderate, interfering with routine activities, moderate level of discomfort; or 3 = severe, unable to perform routine activities, significant level of discomfort. Vaccine-related AEs (possibly related or definitely related) were those that the investigator judged as having a reasonable possibility that the vaccine contributed to the AE. All solicited local AEs were considered vaccine-related. Pre-specified halting/holding rules applied towards the decision on vaccine dose escalation included clinical systemic Treatment-Emergent AEs (vaccine-related Grade 3 Treatment-Emergent AEs beginning within 2 days following vaccination and persisting at Grade 3 for greater than 48 h), laboratory systemic Treatment-Emergent AEs (vaccine-related Grade 3 abnormalities beginning within 3 days following vaccination and persisting at Grade 3 for greater than 48 h), systemic Treatment-Emergent AEs (acute allergic reaction or anaphylactic shock following the administration of vaccine), positive urine pregnancy test, and a vaccine-related serious AE.

### 2.6 Immunogenicity assessments

For immunogenicity assessments, sera were collected on Study Days 0 (pre-vaccination), 28 (1-month post primary vaccination), 56 (pre-vaccination), 84 (1-month post 2nd vaccination), 168 (pre-vaccination) and 196 (1-month post 3rd vaccination). To assess the anti-Pfs25 IgG responses at these time points, an ELISA was conducted on the serum samples. In this assay, 96-well plates were coated with 2 µg/mL of plant-produced Pfs25 antigen (Pfs25MF1E) [28]. Serially diluted test serum samples, starting at a 1:100 dilution, were added to the plates. Antibodies bound to the immobilized Pfs25 antigen were detected using horseradish peroxidase-conjugated goat anti-human IgG and *o*-Phenylenediamine dihydrochloride (SIGMAFAST OPD, Sigma-Aldrich). After enzyme-substrate reaction was stopped by addition of 5 M sulfuric acid solution, the plates were read at 490 nm with 650 nm as a reference using Spectramax M2 microplate reader (Molecular Devices). The endpoint titer of each test sample was calculated as the reciprocal dilution at an optical density (OD) of 1 determined by a 4-parameter curve fit using SoftMax Pro v.5.3 (Molecular Devices). Titers below the limit of quantification (LOQ) were assigned a value of 10.

TRA of the Pfs25 VLP-FhCMB vaccine-induced anti-Pfs25 antibody was measured by the SMFA, by evaluating the development of oocysts in laboratory-reared mosquitos fed IgG antibodies purified from serum, mixed with a red blood cell suspension containing cultured malaria parasites [33]. Pre-immune controls were run in every assay and the reduction in the number of oocysts for each test sample was calculated as a comparison to the corresponding pre-immune control samples. In the presence of functional antibody, the development of oocysts is inhibited, thus the reduction in the number of oocysts compared to the control is a measure of a sample’s TRA. Each IgG sample was tested at 3.75 mg/mL in two independent assays, and if the two data sets did not match (i.e., 95% confidence intervals of % TRA did not overlap) a third assay was conducted. For selected samples, IgGs were tested at 15 mg/mL in a single assay. The SMFA was performed at the Laboratory of Malaria and Vector Research, NIAID, NIH, Rockville, MD.

The immunologic criterion for study continuation and the Go or No-Go decision were based on TRA showing ≥80% reduction in oocysts in ≥50% sera obtained 28 days post 3rd vaccination (Study Day 196) in either Group 3 or 4 subjects compared to the pre-vaccination sera.

### 2.7 Statistical analysis

Statistical analyses were performed by MacroStat Inc. on a final adjudicated locked database using Statistical Analysis Software (SAS)® version 9.3. The primary safety and reactogenicity outcomes included all subjects who received at least one dose of Pfs25 VLP-FhCMB and for whom safety data were available. For extent of exposure, the number and percent of subjects were tabulated by the number of doses received. The number and percent of subjects experiencing at least one solicited AE were summarized by event term (e.g. pain, redness, swelling, fever, etc.). The percent of subjects with at least one event was compared between each vaccine treatment group using Fisher’s exact test. The solicited AEs were also summarized for each post-vaccination period and study day after each vaccination (Days 0–7) by event term and intensity grade for each vaccination period and overall, respectively. If a subject had multiple events occurring in the same event term, the event with the highest severity was counted.

All AEs were coded and summarized by system organ class and preferred term using the Medical Dictionary for Drug Regulatory Activities (MedDRA) Dictionary (version 16.0). If a subject had multiple events occurring in the same body system or same preferred term, the event with the highest severity and the event with the strongest relatedness was counted for the summary of AEs by severity and by relatedness, respectively. No statistical inference between the vaccine treatments was performed on AEs.

For hematology and clinical chemistry, descriptive statistics as well as change from baseline for each test was presented by vaccine treatment group and each visit. Mean changes among vaccine treatment groups were analyzed using an analysis of covariance (ANCOVA) model with an effect for vaccine treatment and baseline value as a covariate for each visit.

Anti-Pfs25 IgG responses assessed by ELISA were analyzed by the non-parametric Friedman test to compare pre- (Study Day 0) versus post-vaccination (Study Days 28, 56, 84, 164 and 196) results. The Dunn’s test was used to adjust *p*-values for multiple comparisons using GraphPad PRISM v.6.02 (GraphPad Software Inc.). The TRA of the test serum samples measured by SMFA was expressed as mean percent inhibition of mean oocyst density (%TRA). The best estimate of %TRA, the 95% CI, and significance of inhibition from two or three feeds were calculated for each IgG as previously described [33], [34] using a zero-inflated negative binomial model. A binomial test was performed to determine whether the %TRA per group per study day was significantly different from no inhibition.

## 3 Results

### 3.1 Study population

Of 44 subjects that were enrolled, all received the 1st scheduled vaccination and all were included in the safety and immunogenicity analyses. Two subjects in each of the 30 μg and 100 μg groups discontinued the study earlier. In the 30 μg group, one subject was “Lost to Follow-up” and another was discontinued because of inability to adhere to the visits schedule; both subjects received all three planned vaccinations. In the 100 μg group, one subject withdrew consent and received only two vaccine doses, and another was discontinued because of “Known illicit and/or intravenous drug abuse” (an exclusion criterion) and only received the first vaccine dose. The subject baseline demographic characteristics are summarized in Table 1. Overall, approximately 60% of participants were male. The mean age of the participants was 34.3 years and the mean weight was 83.74 kg. The majority of the participants (63.6%) were Black or African American.

**Table 1:** Demographic characteristics of Pfs25 VLP-FhCMB Phase 1 trial.

	2 µg (N = 6)	10 µg (N = 6)	30 µg (N = 16)	100 µg (N = 16)	Total (N = 44)
*Gender, n (%)*					
Male	3 (50.0%)	3 (50.0%)	9 (56.3%)	11 (68.8%)	26 (59.1%)
Female	3 (50.0%)	3 (50.0%)	7 (43.8%)	5 (31.3%)	18 (40.9%)

*Age, years*					
Mean (SD)	36.8 (8.93)	36.7 (6.59)	36.1 (8.96)	30.6 (8.86)	34.3 (8.82)
Median	38.0	36.0	35.5	26.5	34.0
Min, Max	25, 47	27, 45	22, 49	18, 49	18, 49

*Ethnicity*					
Non-Hispanic or Latino	4 (66.7%)	5 (83.3%)	15 (93.8%)	14 (87.5%)	38 (86.4%)
Hispanic or Latino	2 (33.3%)	1 (16.7%)	1 (6.3%)	1 (6.3%)	5 (11.4%)
Not reported	0 (0.0%)	0 (0.0%)	0 (0.0%)	1 (6.3%)^1^	1 (2.3%)^1^

*Race, n (%)*					
American Indian or Alaska native	0 (0.0%)	0 (0.0%)	0 (0.0%)	0 (0.0%)	0 (0.0%)
Asian	0 (0.0%)	1 (16.7%)	0 (0.0%)	0 (0.0%)	1 (2.3%)
Black or African-American	4 (66.7%)	3 (50.0%)	10 (62.5%)	11 (68.8%)	28 (63.6%)
White	2 (33.3%)	2 (33.3%)	6 (37.5%)	5 (31.3%)	15 (34.1%)

Percentages are calculated as % = 100 * n/N; SD = Standard Deviation, Min = Minimum, Max = Maximum.

1 One subject in the 100 µg dose group preferred not to provide a response regarding ethnicity.

### 3.2 Safety and reactogenicity

Supplementary data associated with this article can be found, in the online version, at https://doi.org/10.1016/j.vaccine.2018.08.033.

Treatment-Emergent AEs occurring during the study period are summarized in Table 2. Overall, approximately 77% of subjects reported at least one AE, and approximately 23% of reported AEs were considered to be vaccination-related. The highest (100 μg) dose group had the greatest percentage of subjects reporting at least one AE (approximately 94%) as well as subjects reporting vaccination-related AEs (N = 5, approximately 31%) (Table 2). Of these 5 subjects, 1 reported injection site pruritus (not reported as a solicited AE; possibly related) and 2 subjects reported muscle spasm (possibly related) within 28 days post vaccination. The subject who reported possibly related injection site pruritus also reported definitely related injection site pruritus that occurred outside the 28-day post vaccination period. Other than in the 100 μg group, there was no clinically meaningful pattern to the incidences of vaccine-related AEs. Across the three 28-day post-vaccination periods, the overall total proportion of subjects reporting at least one AE, as well as the percentage of subjects reporting vaccination-related AEs, tended to decrease (Supplementary material, Table S1).

**Table 2:** Treatment-Emergent Adverse Events (AEs) that occurred during the study period and Summary of systemic Treatment-Emergent AEs after any vaccination, by intensity grade.^1^

Parameter, n (%)^2^	2 µg (N = 6)	10 µg (N = 6)	30 µg (N = 16)	100 µg (N = 16)	Total (N = 44)
*Study overall*					
Subjects with at least one AE	4 (66.7%)	4 (66.7%)	11 (68.8%)	15 (93.8%)	34 (77.3%)
Related to vaccination	1 (16.7%)	1 (16.7%)	3 (18.8%)	5 (31.3%)	10 (22.7%)
Serious	0 (0.0%)	0 (0.0%)	0 (0.0%)	1 (6.3%)	1 (2.3%)
Outcome of death	0 (0.0%)	0 (0.0%)	0 (0.0%)	0 (0.0%)	0 (0.0%)
Caused discontinuation	0 (0.0%)	0 (0.0%)	0 (0.0%)	0 (0.0%)	0 (0.0%)

*Unsolicited AEs*					
Subjects with at least one AE	4 (66.7%)	4 (66.7%)	11 (68.8%)	15 (93.8%)	34 (77.3%)
Grade 1	1 (16.7%)	0 (0.0%)	6 (37.5%)	10 (62.5%)	17 (38.6%)
Grade 2	3 (50.0%)	4 (66.7%)	5 (31.3%)	5 (31.3%)	17 (38.6%)

*Solicited systemic AEs*					
Subjects with at least one AE	2 (33.3%)	4 (66.7%)	8 (50.0%)	10 (62.5%)	24 (54.5%)
Grade 1	0 (0.0%)	2 (33.3%)	5 (31.3%)	5 (31.3%)	12 (27.3%)
Grade 2	2 (33.3%)	2 (33.3%)	3 (18.8%)	3 (18.8%)	10 (22.7%)
Grade 3	0 (0.0%)	0 (0.0%)	0 (0.0%)	2 (12.5%)	2 (4.5%)

*Solicited local AEs*					
Subjects with at least one AE	4 (66.6%)	6 (100.0%)	12 (75.0%)	15 (93.7%)	37 (84.1%)
Grade 1	1 (16.6%)	4 (66.6%)	12 (75.0%)	4 (25.0%)	21 (47.7%)
Grade 2	3 (50.0%)	2 (33.3%)	0 (0.0%)	11 (68.7%)	16 (36.3%)

Only Treatment-emergent AEs were summarized; i.e., those that started on or after the date/time of the first dose of study vaccine or that worsened on or after the date/time of the first dose, through 28 days post-vaccination. All AEs with onset >28 days post-vaccination are not included in the 28-day post-vaccination summaries but are included in the overall summary (e.g. Serious AE).

Note: “N” for each vaccination may be smaller than that for Full Analysis Set; percentages are calculated as % = n/N.

1 Grading of intensity of AEs: Grade 1 = mild, Grade 2 = moderate, Grade 3 = severe.

2 A subject is counted once in the most severe category if the subject reported one or more events in each event term, but of different intensity.

**Supplementary Tables S1–S3:** 

None of the Treatment-Emergent AEs resulted in death or study discontinuation (Table 2). However, there were 2 Serious AEs: a breast abscess in the 10 μg group and a foot deformity in the 100 μg group (Table 2). The SAE in the 10 μg group occurred >28 days after the last vaccine dose and is therefore not included in Table 2. Both Serious AEs were considered to be unrelated to the study vaccine, and both subjects completed the study and were included in all analyses.

Unsolicited and solicited Treatment-Emergent AEs are summarized by severity in Table 2. Overall, half of the subjects reported at least one Grade 1 Treatment-Emergent AE and half reported at least one Grade 2 Treatment-Emergent AE. In general, subjects in the 2 and 10 μg dose groups reported Grade 2 Treatment-Emergent AEs, while subjects in the 30 and 100 μg dose groups reported Grade 1 events. This pattern was generally consistent during the 28 days after each of the three vaccinations. Within the 28 days after each of the three vaccinations, no more than one subject reported a specific Treatment-Emergent AE that was considered Grade 2 (Supplementary material, Table S1).

Overall, approximately 55% of subjects reported at least one solicited systemic Treatment-Emergent AE, and 22 of these 24 subjects (approximately 90%) had Treatment-Emergent AEs of Grades 1 or 2 (Table 2). The two subjects reporting a Grade 3 Treatment-Emergent AE were both in the 100 μg group (Table 2); one reported fatigue/malaise after the first vaccination and another reported diarrhea after the third vaccination (Supplementary material, Table S3).

The highest incidences of solicited systemic AEs after any vaccination were fatigue/malaise (∼33%), myalgia (∼33%) and chills (∼33%) in the 2 μg dose group; fatigue/malaise (∼33%) and headache (50%) in the 10 μg dose group; fatigue/malaise (25%) and headache (∼31%) in the 30 μg dose group; and headache (25%), nausea (25%), myalgia (∼31%) and fatigue/malaise (∼38%) in the 100 μg dose group.

Overall, approximately 84% of subjects reported at least one solicited local TEAE, and 100% of that 84% had TEAEs considered Grades 1 or 2 (Table 2). Local solicited Treatment-Emergent AEs (injection site reactions) are summarized by event term and severity in Table 3. Pain and tenderness were the only injection site reactions reported among all subjects, and tenderness was the one most frequently reported. Most of these injection site reactions were considered mild discomfort to touch or did not interfere with activity. None of the changes occurring in hematology or clinical chemistry parameters during the study were considered clinically meaningful, demonstrating no safety concerns with any dose group (data not shown).

**Table 3:** Summary of solicited injection site reactions after any vaccination, by event term and intensity grade.^1^

Event term, n (%)^2^	2 µg (N = 6)	10 µg (N = 6)	30 µg (N = 16)	100 µg (N = 16)	Total (N = 44)
Pain	3 (50.0)	3 (50.0)	8 (50.0)	11 (68.7)	25 (56.8)
Grade 1	2 (33.3)	2 (33.3)	8 (50.0)	9 (56.2)	21 (47.7)
Grade 2	1 (16.6)	1 (16.6)	0 (0.0%)	2 (12.5)	4 (9.1)
Tenderness	4 (66.6)	6 (100.0)	12 (75.0)	13 (81.2)	35 (79.5)
Grade 1	1 (16.6)	4 (66.6)	12 (75.0)	3 (18.7)	20 (45.4)
Grade 2	3 (50.0)	2 (33.3)	0 (0.0%)	10 (62.5)	15 (93.7)

1 Grading of intensity of AEs: Grade 1 = mild, Grade 2 = moderate.

2 Subject was counted only once for each event.

### 3.3 Immunogenicity

**Fig. 1:**
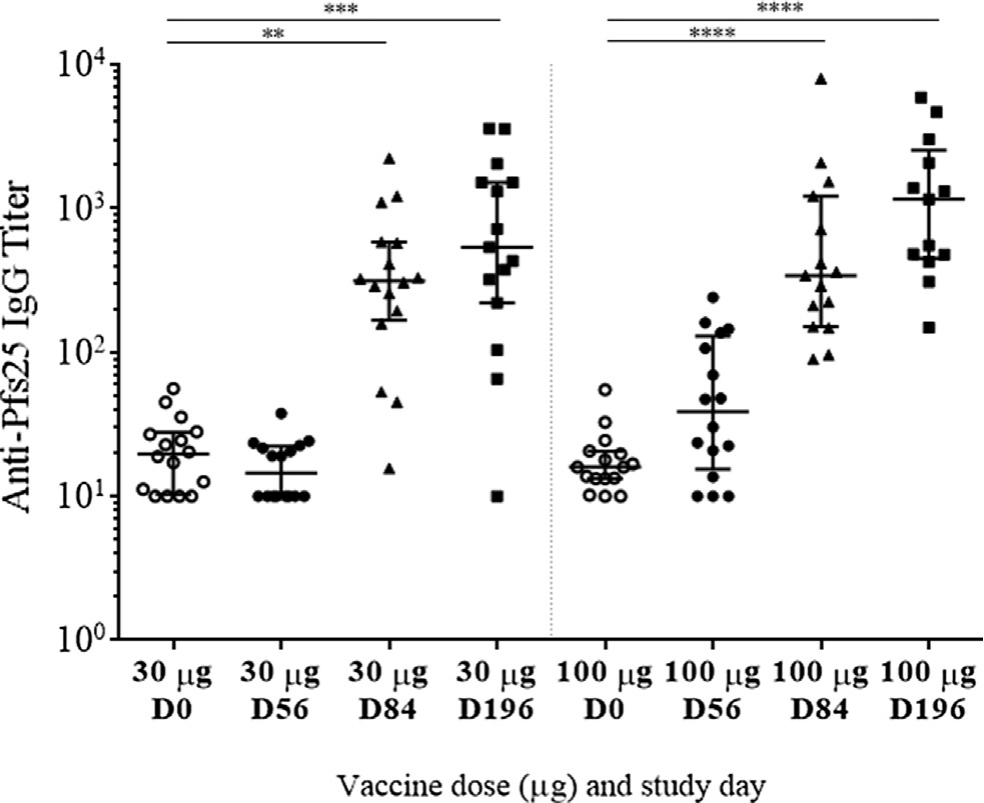
Anti-Pfs25 IgG responses in serum samples collected from the 30 and 100 µg dose groups. Data are shown as median with interquartile range. **: *p* < 0.01, ***: *p* < 0.001, and ****: *p* < 0.0001 when compared to pre-immune data using the Friedman test followed by the Dunn’s multiple comparison test.

There was no significant increase in anti-Pfs25 IgG titers as measured by ELISA after vaccination in the 2 or 10 µg dose groups when compared to the pre-vaccination level (data not shown), except for 1 month after the 3rd vaccination (Study Day 196) in the 10 µg dose group. In the 30 µg dose group, statistically significant increases in anti-Pfs25 IgG titers, as compared to pre-vaccination levels, were observed 1 month after the 2nd (Day 84, *p* < 0.01) and 3rd (Day 196, *p* < 0.001) vaccinations (Friedman test followed by the Dunn’s multiple comparison test, Fig. 1). In the 100 µg dose group, statistically significant increases in anti-Pfs25 IgG titers, as compared to pre-vaccination levels, were observed on Study Days 84 and 196, 1 month after each vaccination (*p* < 0.0001, Fig. 1).

**Fig. 2:**
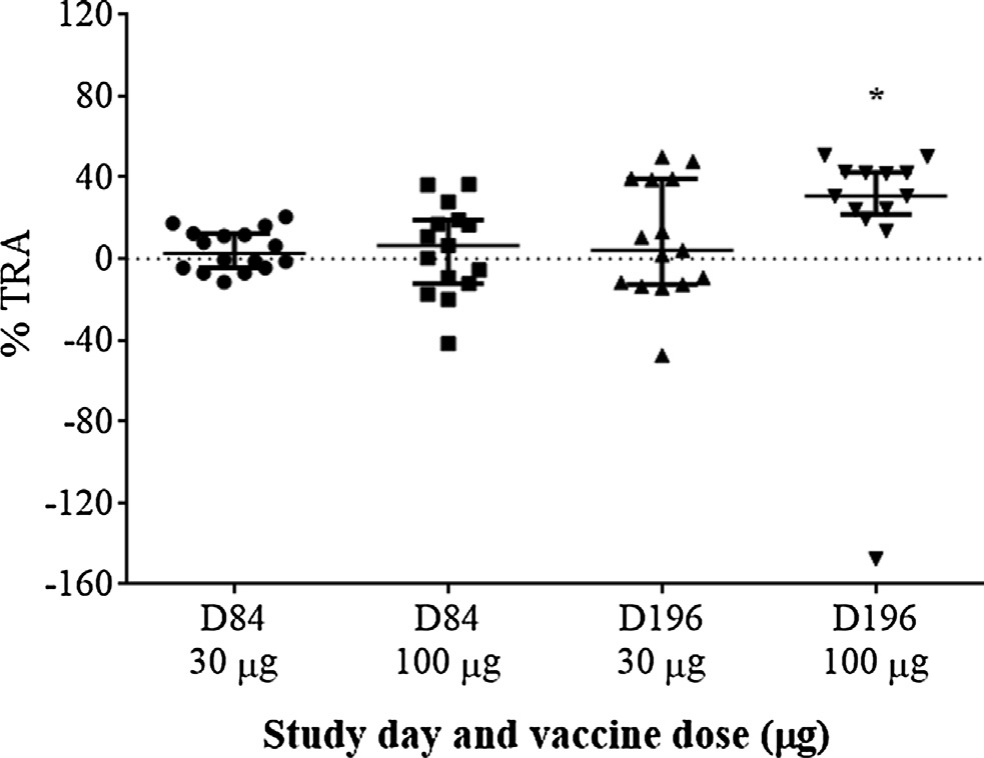
Results of SMFA on sera collected from the 30 and 100 µg dose groups on Study Days 84 (1-month post 2nd dose) and 196 (1-month post 3rd dose). Purified IgG was tested at 3.75 mg/mL in the assay. Data are shown as median with interquartile range. Results are shown as the best estimates from 2 or 3 independent SMFA. *: *p* < 0.05 by the binomial test.

TRA assessment showed that none of the dose groups demonstrated a pre-determined target of ≥80% reduction in oocysts 1 month after the 2nd (Day 84) or 3rd (Day 196) vaccination (Fig. 2). Due to the undetectable or low anti-Pfs25 antibody titers 1-month post 2nd and 3rd vaccination in the 2 μg and 10 μg dose groups, TRA activity was not evaluated in these groups. Both 30 and 100 μg dose groups did not show significant TRA activity after the 2nd vaccination (*p* = 0.598 and 0.304 by binomial tests, respectively), when purified IgG was tested at 3.75 mg/mL. While the 30 μg dose group showed insignificant inhibition post 3rd dose (*p* = 0.607), the 100 µg dose group showed a weak (36.2% on median), but significant, inhibition as a group (*p* = 0.002). However, no subject generated greater than or equal to 80% TRA throughout the study period when tested at IgG concentrations of 3.75 mg/mL. Selected Day 196 samples (5 from the 30 µg and 8 from the 100 μg dose group) were further tested at higher concentration, 15 mg/mL, in SMFA. In these assays, 2 out of the 8 patient samples tested from the 100 µg dose group, had significant %TRA values near 80%: 81% and 77%, respectively.

## 4 Discussion

This first-in-human, Phase 1 clinical study assessed safety and immunogenicity of the Pfs25 VLP-FhCMB, the plant-produced recombinant Pfs25 transmission blocking vaccine candidate against malaria developed by FhCMB for the prevention of disease caused by *P. falciparum*. FhCMB engineered and transiently produced Pfs25 VLP-FhCMB in *N. benthamiana* and subsequently scaled it up for cGMP manufacturing in FhCMB’s pilot facility [32].

At the doses tested in this Phase 1 study, the vaccine was generally shown to be safe in healthy volunteers, with no incidence of vaccine-related Serious AEs and no evidence of any dose-limiting or dose-related toxicity. None of the AEs resulted in a death or discontinuation. As expected, the 100 μg dose group had the greatest percentage of subjects reporting vaccination-related AEs (approximately 31%). Further, across the three 28-day post-vaccination periods the overall total proportion of subjects reporting at least one AE tended to decrease as did the percentage of subjects reporting vaccination-related AEs. The most frequent solicited systemic AEs across the dose groups were fatigue/malaise, headache and myalgia; the only injection site reactions were pain and tenderness AEs, most of which were considered mild. Evaluation of laboratory assessments and vital signs were unremarkable and resulted in no adverse events reported.

Immune analysis of serum samples revealed that the vaccine elicited a good antibody response at total protein doses greater than 30 µg but a weak TRA response by SMFA. A contributing factor to the weak TRA could be the use of Alhydrogel adjuvant in the vaccine formulation. Therefore, the incorporation of a stronger, more potent adjuvant that has a history of safety in humans could enhance the efficacy of the Pfs25 VLP-FhCMB vaccine. In addition, the lack of robust functional immune responses may be due to a suboptimal dose of the target antigen, Pfs25, in the vaccine. The doses used in this study were based on total protein values, of which the majority is the CP from the alfalfa mosaic virus leaving the actual Pfs25 content around 1/10 of the total dose amount. This is less than would be predicted from prior preparations at smaller scale, where 20–30% of CP molecules carried Pfs25 [32]. Therefore, dosing based on Pfs25 content or reengineering of the particle to increase Pfs25 content could also enhance the TRA of the vaccine.

In conclusion, the study was successful in demonstrating an acceptable safety, reactogenicity and tolerability profile of the plant-derived Pfs25 VLP-FhCMB vaccine. There were no halts in dose escalation for this trial. Although the trial did not meet the TRA criterion in the two higher dose groups, the 100 µg dose did demonstrate a weak, but significant, TRA after the 3rd vaccination, suggesting the potential of this vaccine to induce functional antibody titers in humans.
