# Supplementary material for: Safety and immunogenicity of a plant-produced Pfs25 virus-like particle as a transmission blocking vaccine against malaria: A Phase 1 dose-escalation study in healthy adults
Source: Vaccine. 2018 Sep 18;36(39):5865–71. doi: 10.1016/j.vaccine.2018.08.033 (PMC6143384; doi:10.1016/j.vaccine.2018.08.033)
Supplement: Supplementary file 1 [file mmc1.docx]

**Supplementary Material**

**Table S1. Incidence of Solicited TEAEs – Overall and Within 28 Days Post-Vaccination**

| **Event Term, n (%)^1^** | **2 μg**  (N=6) | **10 μg**  (N=6) | **30 μg**  (N=16) | **100 μg**  (N=16) | **Total**  (N=44) | **P-value**  **[1]** |  |
| --- | --- | --- | --- | --- | --- | --- | --- |
| **Study Overall**  Subject with a Least One AE  Fatigue/Malaise  Headache  Myalgia  Nausea  Arthralgia  Chills  Diarrhea  Sweats  Vomiting  Fever | 2 (33.3%)  2 (33.3%)  0 (0.0%)  2 (33.3%)  1 (16.7%)  1 (16.7%)  2 (33.3%)  1 (16.7%)  0 (0.0%)  1 (16.7%)  1 (16.7%) | 4 (66.7%)  2 (33.3%)  3 (50.0%)  1 (16.7%)  1 (16.7%)  0 (0.0%)  1 (16.7%)  0 (0.0%)  1 (16.7%)  0 (0.0%)  0 (0.0%) | 8 (50.0%)  4 (25.0%)  5 (31.3%)  1 (6.3%)  2 (12.5%)  2 (12.5%)  0 (0.0%)  0 (0.0%)  0 (0.0%)  0 (0.0%)  0 (0.0%) | 10 (62.5%)  6 (37.5%)  4 (25.0%)  5 (31.3%)  4 (25.0%)  3 (18.8%)  3 (18.8%)  3 (18.8%)  2 (12.5%)  2 (12.5%)  0 (0.0%) | 24 (54.5%)  14 (31.8%)  12 (27.3%)  9 (20.5%)  8 (18.2%)  6 (13.6%)  6 (13.6%)  4 (9.1%)  3 (6.8%  3 (6.8%)  1 (2.3%) | 0.610  0.963  0.316  0.254  0.936  0.927  0.120  0.234  0.478  0.478  0.273 |  |
| **1st Vaccination- 28 Days Post**  Subjects with at Least One AE  Fatigue/Malaise  Myalgia  Arthralgia  Diarrhea  Headache  Chills  Nausea  Fever  Sweats  Vomiting | 2 (33.3%)  1 (16.7%)  2 (33.3%)  1 (16.7%)  0 (0.0%)  0 (0.0%)  1 (16.7%)  0 (0.0%)  1 (16.7%)  0 (0.0%)  0 (0.0%) | 0 (0.0%)  0 (0.0%)  0 (0.0%)  0 (0.0%)  0 (0.0%)  0 (0.0%)  0 (0.0%)  0 (0.0%)  0 (0.0%)  0 (0.0%)  0 (0.0%) | 4 (25.0%)  2 (12.5%)  1 (6.3%)  1 (6.3%)  0 (0.0%)  2 (12.5%)  0 (0.0%)  0 (0.0%)  0 (0.0%)  0 (0.0%)  0 (0.0%) | 9 (56.3%)  4 (25.0%)  2 (12.5%)  2 (12.5%)  3 (18.8%)  1 (6.3%)  1 (6.3%)  2 (12.5%)  0 (0.0%)  0 (0.0%)  0 (0.0%) | 15 (34.1%)  7 (15.9%)  5 (11.4%)  4 (9.1%)  3 (6.8%)  3 (6.8%)  2 (4.5%)  2 (4.5%)  1 (2.3%)  0 (0.0%)  0 (0.0%) | 0.082  0.776  0.338  0.894  0.174  1.000  0.476  0.729  0.273  1.000  1.000 |  |
| **2nd Vaccination - 28 Days Post**  Subjects with at Least One AE  Headache  Fatigue/Malaise  Myalgia  Arthralgia  Nausea  Chills  Diarrhea  Sweats  Vomiting  Fever | 1 (16.7%)  0 (0.0%)  0 (0.0%)  1 (16.7%)  1 (16.7%)  0 (0.0%)  0 (0.0%)  1 (16.7%)  0 (0.0%)  0 (0.0%)  0 (0.0%) | 1 (16.7%)  1 (16.7%)  0 (0.0%)  0 (0.0%)  0 (0.0%)  0 (0.0%)  0 (0.0%)  0 (0.0%)  0 (0.0%)  0 (0.0%)  0 (0.0%) | 4 (25.0%)  3 (18.8%)  1 (6.3%)  1 (6.3%)  1 (6.3%)  0 (0.0%)  0 (0.0%)  0 (0.0%)  0 (0.0%)  0 (0.0%)  0 (0.0%) | 5 (33.3%)  1 (6.7%)  3 (20.0%)  3 (20.0%)  1 (6.7%)  3 (20.0%)  1 (6.7%)  0 (0.0%)  1 (6.7%)  1 (6.7%)  0 (0.0%) | 11 (25.6%)  5 (11.6%)  4 (9.3%)  4 (9.3%)  3 (7.0%)  3 (7.0%)  1 (2.3%)  1 (2.3%)  1 (2.3%)  1 (2.3%)  0 (0.0%) | 0.922  0.590  0.487  0.234  0.710  0.174  1.000  0.273  1.000  1.000  1.000 |  |
| **3rd Vaccination - 28 Days Post**  Subjects with at Least One AE  Fatigue/Malaise  Headache  Nausea  Arthralgia  Diarrhea  Myalgia  Chills  Sweats | 0 (0.0%)  0 (0.0%)  0 (0.0%)  0 (0.0%)  0 (0.0%)  0 (0.0%)  0 (0.0%)  0 (0.0%)  0 (0.0%) | 2 (33.3%)  2 (33.3%)  2 (33.3%)  1 (16.7%)  0 (0.0%)  0 (0.0%)  1 (16.7%)  0 (0.0%)  0 (0.0%) | 3 (18.8%)  1 (6.3%)  1 (6.3%)  1 (6.3%)  0 (0.0%)  0 (0.0%)  0 (0.0%)  0 (0.0%)  0 (0.0%) | 4 (28.6%)  3 (21.4%)  2 (14.3%)  1 (7.1%)  2 (14.3%)  2 (14.3%)  1 (7.1%)  1 (7.1%)  1 (7.1%) | 9 (21.4%)  6 (14.3%)  5 (11.9%)  3 (7.1%)  2 (4.8%)  2 (4.8%)  2 (4.8%)  1 (2.4%)  1 (2.4%) | 0.597  0.242  0.338  0.710  0.729  0.729  0.476  1.000  1.000 |  |
| ^1^ If a subject experienced the same event more than once, only the occurrence with the highest degree of  categorical relevance is tabulated.  Note: “N” for each vaccination may be smaller than that for Full Analysis Set. Percentages are calculated as  %=n/N. Solicited Event Terms included: pain at the injection site, tenderness at the injection site, erythema at  injection site, induration /swelling at injection site, fever, myalgia, chills, sweats, fatigue/malaise, arthralgia,  headache, nausea, vomiting or diarrhea.  TEAE = treatment-emergent adverse events. Only TEAEs were summarized; i.e., those that started on or after  the date/time of the first dose of study vaccine or that worsened on or after the date/time of the first dose,  through 28 days post-vaccination. All AEs with onset >28 days post-vaccination are not included in the  analysis.  [1] P-values are calculated from a Fisher’s exact test. | | | | | | | |

**Table S2. Incidence of Subjects Reporting at Least One AE with a Mild or Greater Severity ─ Overall and Within 28 Days Post-Vaccination**

| **Parameter, n (%)**^1^ | **2 μg**  (N=6) | **10 μg**  (N=6) | **30 μg**  (N=16) | **100 μg**  (N=16) | **Total**  (N=44) |
| --- | --- | --- | --- | --- | --- |
| **1st Vaccination - within 28 Days** |  |  |  |  |  |
| Abdominal pain upper  Mild  Moderate | 1 (16.7%)  0 (0.0%)  1 (16.7%) | 0 (0.0%)  0 (0.0%)  0 (0.0%) | 0 (0.0%)  0 (0.0%)  0 (0.0%) | 0 (0.0%)  0 (0.0%)  0 (0.0%) | 1 (2.3%)  0 (0.0%)  1 (2.3%) |
| Diarrhea  Mild  Moderate | 1 (16.7%)  0 (0.0%)  1 (16.7%) | 0 (0.0%)  0 (0.0%)  0 (0.0%) | 0 (0.0%)  0 (0.0%)  0 (0.0%) | 0 (0.0%)  0 (0.0%)  0 (0.0%) | 1 (2.3%)  0 (0.0%)  1 (2.3%) |
| Gastroenteritis  Mild  Moderate | 0 (0.0%)  0 (0.0%)  0 (0.0%) | 0 (0.0%)  0 (0.0%)  0 (0.0%) | 1 (6.3%)  0 (0.0%)  1 (6.3%) | 0 (0.0%)  0 (0.0%)  0 (0.0%) | 1 (2.3%)  0 (0.0%)  1 (2.3%) |
| Sinusitis  Mild  Moderate | 0 (0.0%)  0 (0.0%)  0 (0.0%) | 0 (0.0%)  0 (0.0%)  0 (0.0%) | 0 (0.0%)  0 (0.0%)  0 (0.0%) | 1 (6.3%)  0 (0.0%)  1 (6.3%) | 1 (2.3%)  0 (0.0%)  1 (2.3%) |
| Gunshot wound  Mild  Moderate | 0 (0.0%)  0 (0.0%)  0 (0.0%) | 0 (0.0%)  0 (0.0%)  0 (0.0%) | 1 (6.3%)  0 (0.0%)  1 (6.3%) | 0 (0.0%)  0 (0.0%)  0 (0.0%) | 1 (2.3%)  0 (0.0%)  1 (2.3%) |
| Muscle spasms  Mild  Moderate | 0 (0.0%)  0 (0.0%)  0 (0.0%) | 0 (0.0%)  0 (0.0%)  0 (0.0%) | 0 (0.0%)  0 (0.0%)  0 (0.0%) | 2 (12.5%)  1 (6.3%)  1 (6.3%) | 2 (4.5%)  1 (2.3%)  1 (2.3%) |
| Uterine leiomyoma  Mild  Moderate | 0 (0.0%)  0 (0.0%)  0 (0.0%) | 1 (16.7%)  0 (0.0%)  1 (16.7%) | 0 (0.0%)  0 (0.0%)  0 (0.0%) | 0 (0.0%)  0 (0.0%)  0 (0.0%) | 1 (2.3%)  0 (0.0%)  1 (2.3%) |
| Headache  Mild  Moderate | 0 (0.0%)  0 (0.0%)  0 (0.0%) | 1 (16.7%)  0 (0.0%)  1 (16.7%) | 0 (0.0%)  0 (0.0%)  0 (0.0%) | 0 (0.0%)  0 (0.0%)  0 (0.0%) | 1 (2.3%)  0 (0.0%)  1 (2.3%) |
| Anxiety  Mild  Moderate | 0 (0.0%)  0 (0.0%)  0 (0.0%) | 0 (0.0%)  0 (0.0%)  0 (0.0%) | 0 (0.0%)  0 (0.0%)  0 (0.0%) | 1 (6.3%)  0 (0.0%)  1 (6.3%) | 1 (2.3%)  0 (0.0%)  1 (2.3%) |
| Dermatitis contact  Mild  Moderate | 0 (0.0%)  0 (0.0%)  0 (0.0%) | 0 (0.0%)  0 (0.0%)  0 (0.0%) | 2 (12.5%)  2 (12.5%)  0 (0.0%) | 1 (6.3%)  0 (0.0%)  1 (6.3%) | 3 (6.8%)  2 (4.5%)  1 (2.3%) |
| **2nd Vaccination - within 28 Days** |  |  |  |  |  |
| Influenza like illness  Mild  Moderate | 1 (16.7%)  0 (0.0%)  1 (16.7%) | 0 (0.0%)  0 (0.0%)  0 (0.0%) | 0 (0.0%)  0 (0.0%)  0 (0.0%) | 0 (0.0%)  0 (0.0%)  0 (0.0%) | 1 (2.3%)  0 (0.0%)  1 (2.3%) |
| Gastroenteritis  Mild  Moderate | 1 (16.7%)  0 (0.0%)  1 (16.7%) | 0 (0.0%)  0 (0.0%)  0 (0.0%) | 0 (0.0%)  0 (0.0%)  0 (0.0%) | 0 (0.0%)  0 (0.0%)  0 (0.0%) | 1 (2.3%)  0 (0.0%)  1 (2.3%) |
| Nasopharyngitis  Mild  Moderate | 0 (0.0%)  0 (0.0%)  0 (0.0%) | 0 (0.0%)  0 (0.0%)  0 (0.0%) | 1 (6.3%)  0 (0.0%)  1 (6.3%) | 0 (0.0%)  0 (0.0%)  0 (0.0%) | 1 (2.3%)  0 (0.0%)  1 (2.3%) |
| Upper respiratory tract infection  Mild  Moderate | 1 (16.7%)  0 (0.0%)  1 (16.7%) | 0 (0.0%)  0 (0.0%)  0 (0.0%) | 0 (0.0%)  0 (0.0%)  0 (0.0%) | 0 (0.0%)  0 (0.0%)  0 (0.0%) | 1 (2.3%)  0 (0.0%)  1 (2.3%) |
| Procedural pain  Mild  Moderate | 0 (0.0%)  0 (0.0%)  0 (0.0%) | 1 (16.7%)  0 (0.0%)  1 (16.7%) | 0 (0.0%)  0 (0.0%)  0 (0.0%) | 0 (0.0%)  0 (0.0%)  0 (0.0%) | 1 (2.3%)  0 (0.0%)  1 (2.3%) |
| Muscle spasms  Mild  Moderate | 0 (0.0%)  0 (0.0%)  0 (0.0%) | 0 (0.0%)  0 (0.0%)  0 (0.0%) | 2 (12.5%)  1 (6.3%)  1 (6.3%) | 0 (0.0%)  0 (0.0%)  0 (0.0%) | 2 (4.7%)  1 (2.3%)  1 (2.3%) |
| Back pain  Mild  Moderate | 0 (0.0%)  0 (0.0%)  0 (0.0%) | 0 (0.0%)  0 (0.0%)  0 (0.0%) | 0 (0.0%)  0 (0.0%)  0 (0.0%) | 1 (6.7%)  0 (0.0%)  1 (6.7%) | 1 (2.3%)  0 (0.0%)  1 (2.3%) |
| **3rd Vaccination - within 28 Days** |  |  |  |  |  |
| Vomiting  Mild  Moderate | 1 (16.7%)  0 (0.0%)  1 (16.7%) | 0 (0.0%)  0 (0.0%)  0 (0.0%) | 0 (0.0%)  0 (0.0%)  0 (0.0%) | 0 (0.0%)  0 (0.0%)  0 (0.0%) | 1 (2.4%)  0 (0.0%)  1 (2.4%) |
| Mastitis  Mild  Moderate | 0 (0.0%)  0 (0.0%)  0 (0.0%) | 1 (16.7%)  0 (0.0%)  1 (16.7%) | 0 (0.0%)  0 (0.0%)  0 (0.0%) | 0 (0.0%)  0 (0.0%)  0 (0.0%) | 1 (2.4%)  0 (0.0%)  1 (2.4%) |
| Headache  Mild  Moderate | 1 (16.7%)  0 (0.0%)  1 (16.7%) | 0 (0.0%)  0 (0.0%)  0 (0.0%) | 0 (0.0%)  0 (0.0%)  0 (0.0%) | 1 (7.1%)  1 (7.1%)  0 (0.0%) | 2 (4.8%)  1 (2.4%)  1 (2.4%) |
| Anxiety  Mild  Moderate | 1 (16.7%)  0 (0.0%)  1 (16.7%) | 0 (0.0%)  0 (0.0%)  0 (0.0%) | 0 (0.0%)  0 (0.0%)  0 (0.0%) | 0 (0.0%)  0 (0.0%)  0 (0.0%) | 1 (2.4%)  0 (0.0%)  1 (2.4%) |
| ^1^ A subject is counted once in the most severe category if the subject reported one or more events in each  system organ class/preferred term, but different severity.  Note: N=xx for each vaccination may be smaller than that for Full Analysis Set. Percentages are  calculated as %=n/N. Preferred terms are sorted by decreasing total frequency. | | | | | |

**Table S3. Incidence of Solicited Adverse Events by Intensity Grade – Overall and Within 28 Days Post-Vaccination**

| **Event Term, n (%)**^1^ | **2 μg**  (N=6) | **10 μg**  (N=6) | **30 μg**  (N=16) | **100 μg**  (N=16) | **Total**  (N=44) |
| --- | --- | --- | --- | --- | --- |
| **Study Overall**  Subjects with at Least One AE  Grade 1  Grade 2  Grade 3  Fatigue/Malaise  Grade 1  Grade 2  Grade 3  Headache  Grade 1  Grade 2  Grade 3  Myalgia  Grade 1  Grade 2  Grade 3  Nausea  Grade 1  Grade 2  Grade 3  Chills  Grade 1  Grade 2  Grade 3  Diarrhea  Grade 1  Grade 2  Grade 3  Sweats  Grade 1  Grade 2  Grade 3  Vomiting  Grade 1  Grade 2  Grade 3  Fever  100.4-101.1°F  101.2-102.0°F  102.1-104°F | 2 (33.3%)  0 (0.0%)  2 (33.3%)  0 (0.0%)  2 (33.3%)  0 (0.0%)  2 (33.3%)  0 (0.0%)  0 (0.0%)  0 (0.0%)  0 (0.0%)  0 (0.0%)  2 (33.3%)  1 (16.7%)  1 (16.7%)  0 (0.0%)  1 (16.7%)  0 (0.0%)  1 (16.7%)  0 (0.0%)  2 (33.3%)  2 (33.3%)  0 (0.0%)  0 (0.0%)  1 (16.7%)  0 (0.0%)  1 (16.7%)  0 (0.0%)  0 (0.0%)  0 (0.0%)  0 (0.0%)  0 (0.0%)  1 (16.7%)  0 (0.0%)  1 (16.7%)  0 (0.0%)  1 (16.7%)  0 (0.0%)  1 (16.7%)  0 (0.0%) | 4 (66.7%)  2 (33.3%)  2 (33.3%)  0 (0.0%)  2 (33.3%)  2 (33.3%)  0 (0.0%)  0 (0.0%)  3 (50.0%)  1 (16.7%)  2 (33.3%)  0 (0.0%)  1 (16.7%)  1 (16.7%)  0 (0.0%)  0 (0.0%)  1 (16.7%)  1 (16.7%)  0 (0.0%)  0 (0.0%)  1 (16.7%)  1 (16.7%)  0 (0.0%)  0 (0.0%)  0 (0.0%)  0 (0.0%)  0 (0.0%)  0 (0.0%)    1 (16.7%)  1 (16.7%)  0 (0.0%)  0 (0.0%)  0 (0.0%)  0 (0.0%)  0 (0.0%)  0 (0.0%)    0 (0.0%)  0 (0.0%)  0 (0.0%)  0 (0.0%) | 8 (50.0%)  5 (31.3%)  3 (18.8%)  0 (0.0%)  4 (25.0%)  3 (18.8%)  1 (6.3%)  0 (0.0%)  5 (31.3%)  4 (25.0%)  1 (6.3%)  0 (0.0%)  1 (6.3%)  1 (6.3%)  0 (0.0%)  0 (0.0%)  2 (12.5%)  2 (12.5%)  0 (0.0%)  0 (0.0%)  0 (0.0%)  0 (0.0%)  0 (0.0%)  0 (0.0%)  0 (0.0%)  0 (0.0%)  0 (0.0%)  0 (0.0%)  0 (0.0%)  0 (0.0%)  0 (0.0%)  0 (0.0%)  0 (0.0%)  0 (0.0%)  0 (0.0%)  0 (0.0%)  0 (0.0%)  0 (0.0%)  0 (0.0%)  0 (0.0%) | 10 (62.5%)  5 (31.3%)  3 (18.8%)  2 (12.5%)  6 (37.5%)  3 (18.8%)  2 (12.5%)  1 (6.3%)  4 (25.0%)  4 (25.0%)  0 (0.0%)  0 (0.0%)  5 (31.3%)  3 (18.8%)  2 (12.5%)  0 (0.0%)  4 (25.0%)  4 (25.0%)  0 (0.0%)  0 (0.0%)  3 (18.8%)  2 (2.5%)  1 (6.3%)  0 (0.0%)  3 (18.8%)  1 (6.3%)  1 (6.3%)  1 (6.3%)  2 (12.5%)  0 (0.0%)  2 (12.5%)  0 (0.0%)  2 (12.5%)  2 (12.5%)  0 (0.0%)  0 (0.0%)  0 (0.0%)  0 (0.0%)  0 (0.0%)  0 (0.0%) | 24 (54.5%)  12 (27.3%)  10 (22.7%)  2 (4.5%)  14 (31.8%)  8 (18.2%)  5 (11.4%)  1 (2.3%)  12 (27.3%)  9 (20.5%)  3 (6.8%)  0 (0.0%)  9 (20.5%)  6 (13.6%)  3 (6.8%)  0 (0.0%)  8 (18.2%)  7 (15.9%)  1 (2.3%)  0 (0.0%)  6 (13.6%)  5 (11.4%)  1 (2.3%)  0 (0.0%)  4 (9.1%)  1 (2.3%)  2 (4.5%)  1 (2.3%)  3 (6.8%)  1 (2.3%)  2 (4.5%)  0 (0.0%)  3 (6.8%)  2 (4.5%)  1 (2.3%)  0 (0.0%)  1 (2.3%)  0 (0.0%)  1 (2.3%)  0 (0.0%) |
| **1st Vaccination - 28 Days Post**  Subjects with at Least One AE  Grade 1  Grade 2  Grade 3 | 2 (33.3%)  1 (16.7%)  1 (16.7%)  0 (0.0%) | 0 (0.0%)  0 (0.0%)  0 (0.0%)  0 (0.0%) | 4 (25.0%)  4 (25.0%)  0 (0.0%)  0 (0.0%) | 9 (56.3%)  6 (37.5%)  2 (12.5%)  1 (6.3%) | 15 (34.1%)  11 (25.0%)  3 (6.8%)  1 (2.3%) |
| **2nd Vaccination - 28 Days Post**  Subjects with at Least One AE  Grade 1  Grade 2  Grade 3 | 1 (16.7%)  1 (16.7%)  0 (0.0%)  0 (0.0%) | 1 (16.7%)  1 (16.7%)  0 (0.0%)  0 (0.0%) | 4 (25.0%)  2 (12.5%)  2 (12.5%)  0 (0.0%) | 5 (33.3%)  4 (26.7%)  1 (6.7%)  0 (0.0%) | 11 (25.6%)  8 (18.6%)  3 (7.0%)  0 (0.0%) |
| **3rd Vaccination - 28 Days Post**  Subjects with at Least One AE  Grade 1  Grade 2  Grade 3 | 0 (0.0%)  0 (0.0%)  0 (0.0%)  0 (0.0%) | 2 (33.3%)  1 (16.7%)  1 (16.7%)  0 (0.0%) | 3 (18.8%)  3 (18.8%)  0 (0.0%)  0 (0.0%) | 4 (28.6%)  2 (14.3%)  1 (7.1%)  1 (7.1%) | 9 (21.4%)  6 (4.3%)  2 (4.8%)  1 (2.4%) |
| ^1^ A subject is counted once in the most severe category if the subject reported one or more events in each event  term, but different intensity. Event terms are sorted by decreasing total frequency.  Note: N=xx for each vaccination may be smaller than that for Full Analysis Set. Percentages are calculated as  %=n/N.  Note: Solicited Event Terms included: pain at the injection site, tenderness at the injection site, erythema at  injection site, induration/swelling at injection site, fever, myalgia, chills, sweats, fatigue/malaise, arthralgia,  headache, nausea, vomiting or diarrhea. | | | | | |
